# Maxillary molar with two palatal roots: Two case reports

**DOI:** 10.4103/0972-0707.62627

**Published:** 2010

**Authors:** R V S Chakradhar Raju, V Chandrasekhar, Chandra Vijay Singh, Srikanth Pasari

**Affiliations:** Department of Conservative Dentistry and Endodontics, Mamata Dental College, Khammam, AP, India

**Keywords:** Anatomical variations, maxillary molars, number of canals and roots

## Abstract

An awareness and understanding of the presence of an additional root and unusual root canal morphology is essential as it determines the successful outcome of endodontic treatment. Aberrations in root canal anatomy are commonly occurring phenomena. A thorough knowledge of basic root canal anatomy and its variation is necessary for successful completion of endodontic treatment. This report points to the importance of looking for additional roots and canals because knowledge of their existence would enable clinician to treat a case successfully that otherwise might end in failure.

## INTRODUCTION

An awareness and understanding of the presence of additional roots and unusual root canal morphology is essential as it determines the successful outcome of endodontic treatment.[1] In spite of all procedural protocol if clinician's miss an additional root or canals it could pose a great challenge and lead to failure of endodontic treatment.

Anatomic characteristic of permanent maxillary molar is generally described as a group of teeth with three roots, one palatal and two buccal. The occurrence of second mesiobuccal canal is a common variation. Beatty reported a first maxillary molar with five root canals, three of them located in mesio buccal root.[2] Bond *et al.* Martinez-Berna, Ruiz-Badanelli studied 338 maxillary first molar's and reported three cases of six canals with three canals in the mesio buccal root two in disto buccal and one in palatal root.[3,4] Christie *et al.* analyzed endodontic treatment in 16 maxillary molars and of six extracted teeth with two palatal roots and classified these 22 molars into three types, according to root separation level and their divergences.[5]

### Case Reports

**Figure 1:**
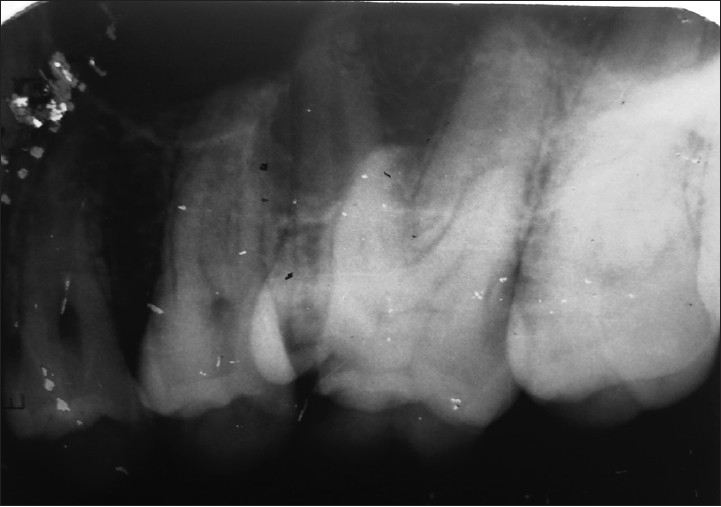
Preoperative radiograph of 26

**Figure 2:**
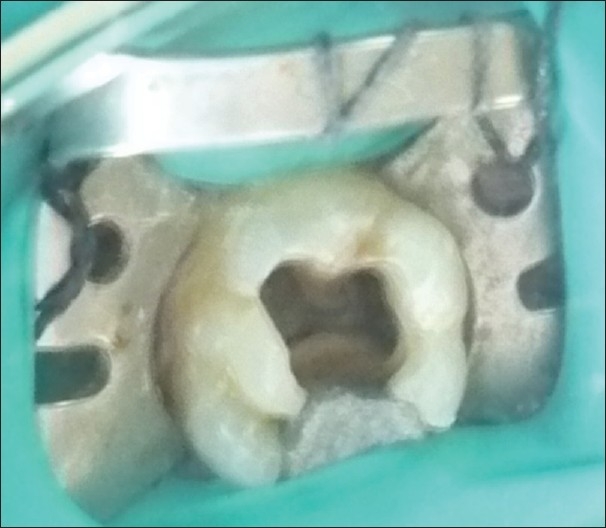
Clinical presentation of 26

**Figure 3:**
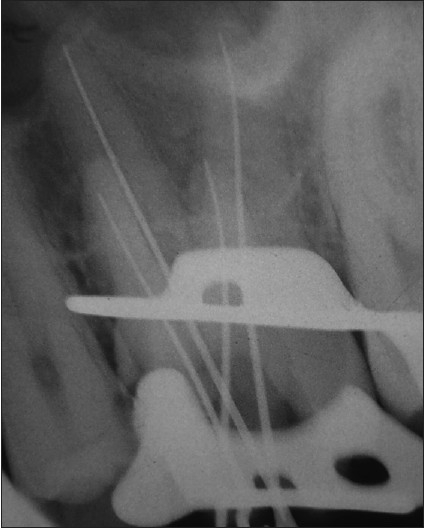
Working length radiograph of 26

**Figure 4:**
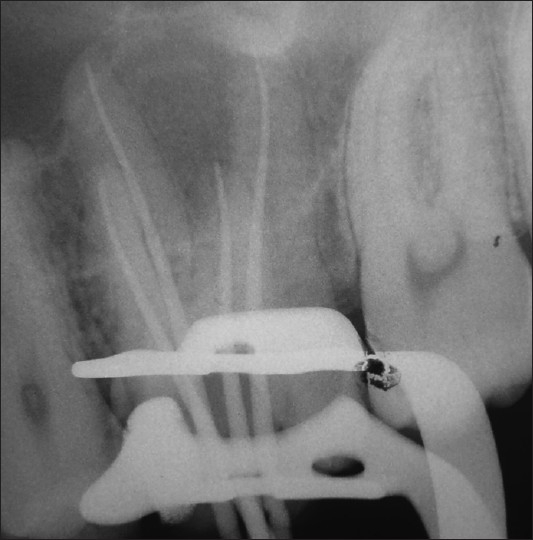
Master cone radiograph of 26

**Figure 5:**
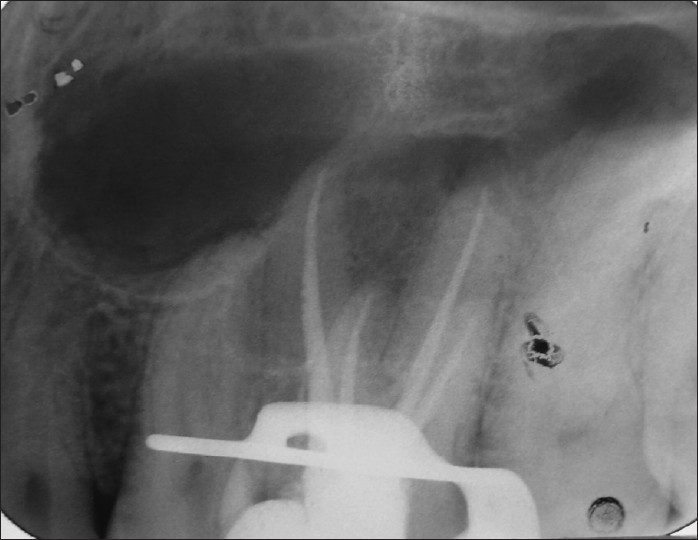
Post obturation radiograph of 26

A 24-year-old male patient with pain in 26 was referred to the Department of Endodontics. Medical history was non contributory. Clinical, radiographic and pulp testing examination revealed that tooth was symptomatic and patient requires endodontic treatment. The preoperative radiograph revealed the presence of an additional palatal root [Figure 1]. The patient was prepared for endodontic treatment and received local anesthesia of 2% lidocaine with 1:80,000 epinephrine. A rubber dam was placed and a conventional endodontic access opening made. After removing the coronal pulp and probing with a DG16 endodontic explorer, three principal root canal orifices - mesiobuccal, distobuccal, palatal and, in addition, a small hemorrhagic point were noted adjacent to the palatal orifice. The conventional triangular access was modified to a trapezoidal shape to improve access to additional canal [Figure 2]. The working length of each canal was estimated by means of an apex locator (Root ZX: Morita, Tokyo, Japan), and confirmed with intra oral periapical radiograph [Figure 3]. The canals were initially instrumented with #15 nickel titanium files (Dentsply Maillefer) under irrigation with 3% sodium hypochlorite. Biomechanical preparation was performed using the crown down technique with nickel-titanium rotary instruments (HERO rotary files, MicroMega, Besancon France). All canals were enlarged to a 30.04 file. Master cone radiograph was taken [Figure 4]. Final irrigation with 17% EDTA followed by 3% sodium hypochlorite and sealing of root canal space with gutta-percha and AH plus resin sealer using lateral condensation technique and tooth was restored with a posterior composite filling [Figure 5].

### Case 2

**Figure 6:**
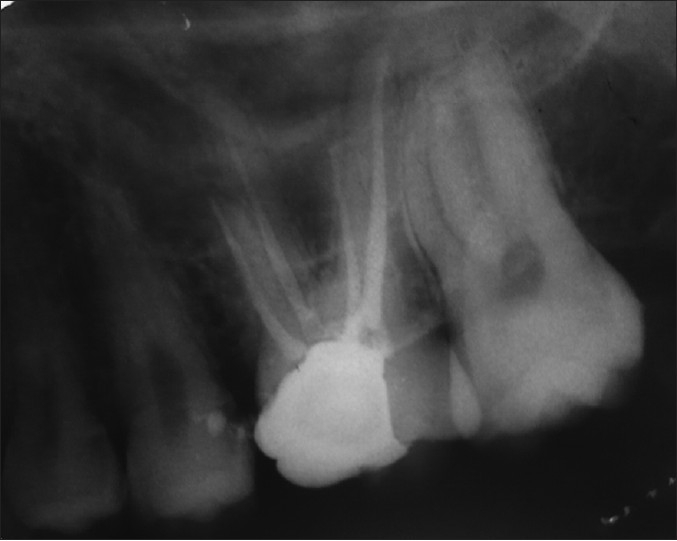
Post obturation radiograph of 16

A 21-year-old male patient was referred to the Department of Endodontics, with pain in 16. Medical history was non contributory. Clinical, radiographic and pulp testing examination revealed that tooth was symptomatic and patient requires endodontic treatment. Anesthetizing the tooth, a conventional endodontic access opening was made. After removing the coronal pulp and probing with a DG 16 three principal root canal orifice mesiobuccal, distobuccal, palatal and in addition a small hemorrhagic point was noted adjacent to the palatal orifice. The conventional triangular access was modified to a trapezoidal shape to improve access to additional canal and by evaluating radiograph from different angulations, it was concluded that this tooth was having an additional palatal root. The working length of each canal was estimated by means of an apex locator (Root ZX: Morita, Tokyo, Japan), and confirmed with intra oral periapical radiograph. The canals were initially instrumented with #15 nickel titanium files (Dentsply Maillefer) under irrigation with 3% sodium hypochlorite and 17% EDTA. Coronal flaring was carried out by using Gates Glidden drills (number 3 and 2 Dentsply Maillefer). Cleaning and shaping were done using hand nickel titanium file system (Dentsply Maillefer). The canals were obturated with AH plus resin sealer and gutta-percha points using lateral condensation technique and tooth was restored with a posterior composite filling [Figure 6].

## DISCUSSION

Anatomical variations can occur in maxillary permanent molars. Christie *et al*.[6] reported 16 cases of maxillary molar with two palatal roots and classified them into three types –

Type 1 – Buccal roots are often cow-horn shaped and less divergent. It has two widely divergent palatal roots, often long and tortuous.

Type 2 – Roots are shorter run parallel and have blunt root apices.

Type 3 – Root morphology is constricted with mesiobuccal, mesiopalatal, distopalatal canal engaged in one web of root dentin. The distobuccal root seems to stand alone and may diverge to distobuccal.

The present two cases come under Type 1 Christie *et al.* classification.

Slowey also reported maxillary molar with two palatal roots.[6] Libfield and Rostein examined 1200 molar and found 0.4% incidence of maxillary molar with four roots[7]. Benenati reported a maxillary second molar with two palatal roots and groove located in this side of the tooth.[8]

The etiology behind formation is unclear. In supernumerary roots its formation could be related to external factors during odontogenesis or penetrance of atavastic gene. Curzon suggests that additional rooted molar trait has high degree of genetic penetrance.[9]

In mandibular molars also there could be variation in root morphology. Carlsen and Alexandersen suggest that if additional root is present distolingually it be termed Radix Entomolaris[10] If additional root is present mesiobucally, it be termed Radix Paramolaris.[11]

To determine for presence of additional root there should be slight different approach besides normal procedural protocol and the clinician should look for following signs which might indicate towards the presence of additional root –

1. Cervical prominence – it could be detected through periodontal probing
2. Extra cusp – which is present in combination with cervical prominence
3. Radiographic examination – radiograph should be taken at different angulations
4. CT scan

## CONCLUSION

The variation in root or root canal morphology, especially in multirooted teeth, is challenging for diagnosis and successful endodontic therapy. The knowledge of common anatomic characteristics and their possible variation is fundamental. Knowledge of unknown variation like the case discussed is essential as non treatment of one additional root or root canal can lead to failure of endodontic treatment. The presence of an additional palatal root is rare but still an unforeseen eventuality and whenever an endodontist is confronted with unusual difficulties and patient complains of persistent post medicament pain, the possibility of an extra canal or root has to be borne in mind. The present report emphasizes the need for the endodontist to be ever vigilant and knowledgeable about aberrant anatomical situations.
